# City-Level Sugar-Sweetened Beverage Taxes and Changes in Adult Body Mass Index

**DOI:** 10.1001/jamanetworkopen.2024.56170

**Published:** 2025-01-24

**Authors:** Emily F. Liu, Deborah R. Young, Margo A. Sidell, Catherine Lee, Deborah A. Cohen, Lee J. Barton, Jennifer Falbe, Galina Inzhakova, Sneha Sridhar, Allison C. Voorhees, Bing Han, Monique M. Hedderson

**Affiliations:** 1 Division of Research, Kaiser Permanente Northern California, Pleasanton; 2 Department of Research and Evaluation, Kaiser Permanente Southern California, Pasadena; 3 Department of Epidemiology and Biostatistics, University of California, San Franscico; 4 Department of Human Ecology, University of California, Davis; 5 Center for Upstream Prevention of Adiposity and Diabetes Mellitus, Division of Research, Kaiser Permanente Northern California, Pleasanton; 6 Kaiser Permanente Bernard J. Tyson School of Medicine, Pasadena, California

## Abstract

**Importance:**

Sugar-sweetened beverage (SSB) excise taxes are popular policy interventions aimed at decreasing SSB purchasing and consumption to improve cardiometabolic health and generate revenue for public health initiatives. There is limited evidence that these taxes in the US are associated with weight-related outcomes in adults, a primary contributor to cardiometabolic health.

**Objective:**

To determine the association between SSB excise taxes and adult body mass index (BMI) and proportion of adults with overweight or obesity among California cities and assess whether associations vary by demographic characteristics.

**Design, Setting, and Participants:**

This cohort study compared California cities with SSB taxes (Albany, Berkeley, Oakland, and San Francisco) and demographically matched cities without SSB excise taxes from 6 years before the tax and 4 to 6 years after the tax from January 2009 through December 2020 using electronic health record data. Participants were Kaiser Permanente (KP) members aged 20 to 65 years at cohort entry with at least 1 pretax and 1 posttax BMI measurement. Data were analyzed from January 2021 to May 2023.

**Exposure:**

Implementation of city-level SSB excise taxes.

**Main outcomes and measures:**

Mean BMI and proportion of adults with overweight or obesity. Analysis used the differences-in-differences (DID) method.

**Results:**

The cohort had a total of 1 044 272 members (178 931 participants in 4 cities with excise taxes; mean [SD] age, 39.7 [11.2] years; 99 501 [55.6%] female; 865 343 participants in 40 control cities without excise taxes; mean [SD] age, 39.9 [11.6] years; 480 155 [55.5%] female). DID estimates for mean BMI showed a modest decrease among adults aged 20 to 39 years (20-25 years: −0.30; 95% CI, −0.51 to −0.08; 26-39 years: −0.19; 95% CI, −0.37 to −0.20), female participants (−0.19; 95% CI, −0.26 to −0.11), and White participants (−0.19; 95% CI, −0.35 to −0.04) living in cities with SSB excise taxes. There was a statistically significant reduction in mean BMI in Berkeley (−0.16; 95% CI, −0.27 to −0.04). There were no overall differences in BMI or proportion with overweight or obesity.

**Conclusions and relevance:**

In this cohort study, SSB excise taxes were associated with reduced mean BMI among adults in demographic subgroups, including in young adults who consumed the most SSBs, and in Berkeley. Future research should examine the mechanisms of these associations to inform how SSB taxes could be more equitable for weight-related outcomes.

## Key Points

**Question:**

Are city-level excise taxes on sugar-sweetened beverages (SSB) associated with changes in adult body mass index (BMI) and proportion of adults with overweight or obesity 4 to 6 years after tax implementation in California, and do associations vary by key demographics?

**Findings:**

In this cohort study of 1 044 272 participants, BMI decreased modestly among adults aged 20 to 39 years, female, or White living in cities with SSB taxes and overall in Berkeley compared with those living in control cities from 2009 to 2020. Overall, there were no differences in BMI or proportion with overweight or obesity.

**Meaning:**

These findings suggest SSB excise taxes were associated with decreased adult BMI among specific demographic groups and in Berkeley.

## Teaser

This cohort study evaluates the association of sugar-sweetened beverage excise taxes with adult body mass index in California cities.

## Introduction

Most adults in the US have overweight (31.1%) or obesity (42.5%), and therefore are at a higher risk of serious and costly cardiometabolic conditions such as type 2 diabetes and cardiovascular disease compared with those with healthy body mass index (BMI).^1^ One cause of overweight and obesity is the consumption of sugar-sweetened beverages (SSB), which are the top source of added sugar in the US diet.^2^ SSBs are calorie-dense but nutrient poor, and consumption of SSBs could result in low levels of satiety, leading to excess calorie consumption and weight gain.^2^

SSB excise taxes (hereafter, SSB taxes) are a policy intervention intended to improve cardiometabolic conditions and generate revenue for public health initiatives.^3^ Taxes are imposed on SSB distributors, who pass on a portion of these taxes to consumers.^4,5^ This results in higher shelf prices, which discourage SSB purchasing and consumption. A recent evaluation^6^ examining 4 US cities with SSB taxes found a one-third increase in SSB prices and a one-third decrease in SSB purchases 2 years after the tax. While decreased purchases may represent lower SSB consumption, evidence of SSB taxes’ association with key health outcomes among adults is still emerging. An assessment^7^ of Seattle’s SSB tax found that adults living in Seattle had a lower annual change in BMI relative to comparison areas 2 years after the tax. In Philadelphia, there was limited evidence of a modest reduction in BMI and obesity prevalence among adults 3 years after the tax.^8^ These findings suggest that SSB taxes may lower BMI among adults, but more research is needed to understand their long-term effects on adult BMI and obesity prevalence. Additionally, verifying these results in other US cities will help policymakers determine whether the benefits of SSB taxes vary depending on how and where they are implemented.

In our prior work,^9^ SSB taxes were associated with overall lower BMI percentiles among youth aged 2 to 19 years. This study extends the cohort to adults aged 20 to 65 years living in California cities with (178 931 participants) and without (865 343 participants) SSB taxes. We used electronic health record (EHR) data from Kaiser Permanente (KP) Northern and Southern California 6 years before tax implementation and 4 to 6 years after to examine the association of SSB taxes on adult BMI and proportion of adults with overweight or obesity and whether associations vary by key demographic characteristics. We hypothesized that cities with SSB taxes would have lower BMI after tax implementation compared with the control cities.

## Methods

This cohort study was conducted at KP from January 2009 through December 2020 and included both youths (aged 2-19 years) and adults (aged 20-65 years). Findings for youths and detailed study methods have been previously published.^9^ KP is an integrated health care delivery system with robust clinical data in its EHR in addition to sociodemographic and geographic information in its membership database. All data were obtained from the EHR from KP’s Northern and Southern California regions. Over this period, both regions combined served over 8.1 million members who were representative of the statewide population and respective regional populations.^10,11^ This study was reviewed and approved by the KP institutional review boards and met the Strengthening the Reporting of Observational Studies in Epidemiology (STROBE) reporting guidelines. Informed consent was waived because the data were deidentified, in accordance with 45 CFR §46.

In California, there were 4 total cities with SSB taxes implemented at 2 separate times: Berkeley in 2015 and Albany, Oakland, and San Francisco in 2017.^3^ Table 1 presents the tax rate and relevant years analyzed in this study by exposure city. Cities without SSB taxes and listed as the primary residential address of KP patients were eligible to be potential controls (328 cities). Cities were matched without replacement using nearest-neighbor Euclidian distance defined by standardized city-level data from the 5-year American Community Survey (ACS) before SSB tax implementation year. Matched exposure and control cities could not share geographic borders because people may not participate in daily activities (eg, shopping or work) in the same city as their primary residence.

**Table 1.** Tax Rate and Years of Implementation and Analysis by Cities With Sugar-Sweetened Beverage (SSB) Excise Taxes

City with SSB excise tax	Tax rate (cent/ounce)	Year of tax implementation	Tax years analyzed	
			Before tax	After tax^a^
Albany	1.00	2017	2011-2016	2017-2020
Berkeley	1.00	2015	2009-2014	2015-2020
Oakland	1.00	2017	2011-2016	2017-2020
San Francisco	1.00	2017	2011-2016	2017-2020

^a^ 2020 was the most recent year available in the data because analysis was conducted from 2021 to 2023.

Matching was assessed through comparison of population-based demographics of each city with an SSB excise tax city and its control cities. Overall, the population total of cities with SSB taxes were notably different than their matched control cities. There were also slight discrepancies in age group, race and ethnicity, and educational attainment. However, cities were similar in distribution of households below the poverty line, sex, and KP membership. Details of the city-matching process are described in the eAppendix, eTables 1 and 2, and eFigure in Supplement 1.

Patients with a primary residential address in the matched cities were included in the analysis if they were aged 20 to 65 years at cohort entry (ie, first pretax BMI measurement), had an active KP membership in at least 1 year during the study period, and had at least 1 BMI measurement before and after tax implementation. We limited the maximum age to 65 years, given that BMI is a poor indicator of adiposity in older adults.^12^ Patients were excluded due to health conditions that may affect BMI, including history of metastatic cancer, bariatric surgery in the last 5 years, currently being pregnant, or currently receiving palliative care. Patients were also excluded if they reported a change in primary residential address in another city.

BMI was calculated as weight in kilograms divided by height in meters squared using measurements from inpatient or outpatient visits. Multiple BMI measurements over the course of a year were averaged to obtain 1 yearly measurement per patient. BMI was used to categorize patients with overweight (25 to <30) and obesity (≥30). We applied an alternative BMI cutoff for overweight (23 to <27.5) and obesity (≥27.5) for patients with Asian ancestry using self-reported race as a proxy for ancestry.^13^ Biologically implausible values for BMI (≤5 or ≥91) were excluded, totaling less than 0.1% of the study population who had at least 1 out-of-range BMI value. Insurance type at cohort entry and race and ethnicity were additional EHR covariates. Insurance type was a proxy for income and socioeconomic status because patients with low-income and fewer resources had public insurance (ie, Medicaid). Race and ethnicity are socially constructed and included in this study given evidence of racial and ethnic differences in SSB consumption^14,15^ and marketing exposure.^16^ Race and ethnicity were self-reported and categorized into 5 groups: non-Hispanic Asian or Pacific Islander (Asian or Pacific Islander), non-Hispanic Black (Black), Hispanic, non-Hispanic White (White), and other races and ethnicities (including non-Hispanic multiracial and non-Hispanic Native American or Alaskan Native). Census-tract level poverty was obtained from 5-year ACS estimates using the patient’s primary residential address the year of SSB tax implementation.

### Statistical Analysis

We used a differences-in-differences method for our analysis.^17^ We leveraged our large sample and used a stratified analytic approach. We tested the parallel trends assumption between the cities with SSB taxes and controls using placebo tests. All contrasts comparing outcomes under 2 exposure distributions and the placebo test statistics were estimable under the general linear hypothesis inference framework.^18^ Overall results grouped all exposure cities together and used a weighted combination of block-level models, weighted by sample sizes of the exposure cities in each block. Stratified effect estimates were assessed for exposure city, racial and ethnic group, age group, sex, years after the tax, and living in a high-poverty area (ie, primary residence in a census tract where 20% or more of households were living below poverty). Analyses were conducted in SAS version 9.4 (SAS Institute). Technical details of the statistical analysis are described in eAppendix in Supplement 1. We applied Wald *z* test to calculate 95% CIs and *P* values for the aggregated results. *P* ≤ .05 was considered statistically significant. Data analysis was performed from January 2021 to May 2023.

## Results

The characteristics of the study cohort are presented in Table 2. The final sample had a total of 1 044 272 members (178 931 participants in 4 cities with SSB taxes; mean [SD] age, 39.7 [11.2] years; 99 501 [55.6%] female; 43 179 [24.1%] Asian or Pacific Islander, 22 521 [12.6%] Black, 29 296 [16.4%] Hispanic, and 73 664 [41.2%] White; 865 343 participants in 40 control cities without SSB taxes; mean [SD] age, 39.9 [11.6] years; 480 155 [55.5%] female; 185 165 [21.4%] Asian or Pacific Islander, 69 069 [8.0%] Black, 274 546 [31.7%] Hispanic, and 287 886 [33.3%] White). At time of cohort entry, more than half of patients had overweight or obesity. Overall, cities with SSB taxes had a notably lower proportion of patients with overweight or obesity compared with control cities (106 695 patients [59.6%] vs 617 617 patients [71.4%], respectively). While the mean age, distribution of sex, and proportion of households living at or below poverty were similar, there were moderate differences in the distribution of racial and ethnic groups. Cities with SSB taxes had a greater proportion of Black (ie, Albany, Berkeley, and Oakland) and White (ie, Berkeley, Oakland, and San Francisco) adults, and fewer Hispanic adults (ie, Albany, Berkeley, Oakland, and San Francisco). Additionally, cities with SSB taxes had fewer patients with Medicaid (ie, Oakland and San Francisco) compared with their respective control cities.

**Table 2.** Characteristics of Exposure and Control Cities for Kaiser Permanente Patients Aged 20 to 65 Years at Baseline (First Year of Cohort Entry)

Characteristic	Patients, No (%)									
	Albany		Berkeley		Oakland		San Francisco		Total^a^	
	Exposure group (n = 2651)	Control group (n = 72 028)	Exposure group (n = 12 505)	Control group (n = 63 352)	Exposure group (n = 63 001)	Control group (n = 274 092)	Exposure group (n = 101 197)	Control group (n = 457 675)	Exposure group (n = 178 931)	Control group (n = 865 343)
Age, mean (SD), y	42.6(10.7)	41.6 (11.1)	41.8 (11.4)	40.2 (11.4)	39.6 (11.1)	39.8 (11.7)	39.4 (11.2)	39.7 (11.7)	39.7 (11.2)	39.9 (11.6)
Race										
Asian or Pacific Islander	739 (27.9)	21 020 (29.2)	1389 (11.1)	15 324 (24.2)	8589 (13.6)	49 203 (18.0)	32 514 (32.1)	100 122 (21.9)	43 179 (24.1)	185 165 (21.4)
Black	102 (3.8)	1870 (2.6)	1281 (10.2)	2653 (4.2)	15 696 (24.9)	39 066 (14.3)	5488 (5.4)	25 644 (5.6)	22 521 (12.6)	69 069 (8.0)
Hispanic	248 (9.4)	8889 (12.3)	1201 (9.6)	16 036 (25.3)	12 772 (20.3)	95 708 (34.9)	15 125 (14.9)	154 426 (33.7)	29 296 (16.4)	274 546 (31.7)
White	1392 (52.5)	35 878 (49.8)	7871 (62.9)	25 567 (40.4)	22 361 (35.5)	73 536 (26.8)	42 293 (41.8)	153 428 (33.5)	73 664 (41.2)	287 886 (33.3)
Other^b^	170 (6.4)	4371 (6.1)	763 (6.1)	3772 (6.0)	3583 (5.7)	16 579 (6.0)	5777 (5.7)	24 055 (5.3)	10 271 (5.7)	48 677 (5.6)
Sex										
Female	1560 (58.8)	39 893 (55.4)	7063 (56.5)	34 603 (54.6)	37 193 (59.0)	156 327 (57.0)	53 969 (53.3)	250 448 (54.7)	99 501 (55.6)	480 155 (55.5)
Male	1091 (41.2)	32 135 (44.6)	5442 (43.5)	28 749 (45.4)	25 808 (41.0)	117 765 (43.0)	47 228 (46.7)	207 227 (45.3)	79 430 (44.4)	385 188 (44.5)
State-subsidized health insurance plan	62 (2.3)	1867 (2.6)	254 (2.0)	1302 (2.1)	2613 (4.1)	20 413 (7.4)	2483 (2.5)	18 646 (4.1)	5389 (3.0)	42 112 (4.9)
Neighborhood household poverty >20%^c^	85 (3.3)	390 (0.6)	1741 (15.3)	2652 (4.8)	21 342 (36.7)	46 097 (18.2)	4873 (5.4)	54 663 (13.6)	27 964 (17.3)	103 662 (13.3)
BMI, mean (SD)^d^	25.8 (5.2)	26.9 (5.5)	26.2 (5.6)	27.5 (6.0)	28.0 (6.6)	29.3 (6.6)	26.1 (5.4)	28.2 (6.2)	26.8 (5.9)	28.3 (6.3)
Overweight^e^	939 (35.4)	27 281 (37.9)	4105 (32.8)	23 387 (36.9)	21 219 (33.7)	95 169 (34.7)	36 250 (35.8)	165 317 (36.1)	62 382 (34.9)	310 506 (35.9)
Obese^f^	520 (19.6)	19 001 (26.4)	2490 (19.9)	18 916 (29.9)	19 545 (31.0)	112 767 (41.1)	21 848 (21.6)	157 058 (34.3)	44 313 (24.8)	307 111 (35.5)
Overweight or obese^g^	1459 (55.0)	46 282 (64.3)	6595 (52.7)	42 303 (66.8)	40 764 (64.7)	207 936 (75.9)	58 098 (57.4)	322 375 (70.4)	106 695 (59.6)	617 617 (71.4)

Abbreviation: BMI, body mass index.

^a^ The total exposure and total controls do not add up to these totals. There were people who qualified to participate in 2 cities and were only included 1 time in the total count.

^b^ Other race refers to American Indian or Alaskan Native, multiracial, and other races and ethnicities not listed or unknown.

^c^ Neighborhood defined as the census tract of a patient’s residence.

^d^ Calculated as weight in kilograms divided by height in meters squared.

^e^ Overweight categorized as a BMI equal to 25 and less than 30.

^f^ Obese categorized as a BMI equal or greater than 30.

^g^ Overweight or obese categorized as BMI equal to or greater than 25.

Table 3 presents the results for the overall and subgroup analyses adjusted for differences in individual and community-level characteristics at the time of cohort entry and secular trends. Across all cities with SSB taxes, the change in mean BMI before and after tax implementation was not significant compared with control cities (−0.13; 95% CI, −0.28 to 0.01). There were also no differences in the change of proportion of adults with overweight or obesity (0.4 percentage points [pp]; 95% CI, −1.2 to 2.0 pp). Associations were also examined stratified by city with SSB tax, age category, sex, race and ethnicity, and year of posttax implementation. SSB taxes were associated with a reduction in mean BMI among adults who were aged between 20 to 39 years at cohort entry (20-25 category: −0.30; 95% CI, −0.51 to −0.08; 26-39 category: −0.19; 95% CI, −0.37 to −0.20), were female (−0.19; 95% CI, −0.26 to −0.11), or were White (−0.19 95% CI, −0.35 to −0.04). In Berkeley, the change in mean BMI before and after tax implementation was statistically significant compared with its control cities (−0.16; 95% CI, −0.27 to −0.04), and was most pronounced in its fifth year of tax implementation (year 5: −0.24; 95% CI, −0.44 to −0.03). There were no differences in the proportion of adults with overweight or obesity in the stratified analyses, except for a slight increase among the high-poverty subgroup (1.5 pp; 95% CI, 0.5 to 2.4 pp).

**Table 3.** Overall and Subgroup Results by the Differences-in-Difference Analysis^a^

Characteristic	Additive differences in mean BMI (95% CI)^b^	Percentage point change in adults with overweight or obesity (95% CI)
Overall	−0.132 (−0.277 to 0.014)	0.4 (−1.2 to 2.0)
City		
Albany	−0.012 (−0.275 to 0.251)	−0.5 (−4.6 to 3.7)
Berkeley	−0.156 (−0.268 to −0.043)^c^	−0.1 (−1.8 to 1.6)
Oakland	−0.100 (−0.200 to 0.001)	0.8 (−1.0 to 2.5)
San Francisco	−0.152 (−0.327 to 0.023)	0.3 (−1.1 to 1.7)
Age, y		
20-25	−0.295 (−0.513 to −0.077)^c^	−1.0 (−3.7 to 1.7)
26-39	−0.194 (−0.369 to −0.020)^c^	0.6 (−1.5 to 2.8)
40-54	−0.110 (−0.242 to 0.022)	0.5 (−1.0 to 1.9)
55-65	−0.045 (−0.172 to 0.083)	0.3 (−1.3 to 1.9)
Sex		
Female	−0.185 (−0.261 to −0.109)^c^	−0.1 (−1.6 to 1.5)
Male	−0.065 (−0.136 to 0.007)	1.1 (−0.6 to 2.7)
Race and ethnicity		
Asian or Pacific Islander	−0.133 (−0.278 to 0.012)	0.4 (−1.7 to 2.5)
Black	−0.094 (−0.206 to 0.019)	−0.3 (−1.6 to 1.0)
Hispanic	−0.054 (−0.223 to 0.115)	0.2 (−1.7 to 2.0)
White	−0.193 (−0.345 to −0.040)^c^	0.7 (−0.5 to 1.9)
Other^d^	−0.006 (−0.115 to 0.103)	0.7 (−1.5 to 2.8)
High poverty cohort^e^	−0.018 (−0.152 to 0.117)	1.5 (0.5 to 2.4)^c^
Posttax implementation year		
1 (tax implementation)^f^	−0.098 (−0.282 to 0.086)	0.3 (−1.8 to 2.4)
2	−0.123 (−0.318 to 0.072)	0.5 (−1.7 to 2.7)
3	−0.156 (−0.363 to 0.051)	0.3 (−2.1 to 2.6)
4	−0.142 (−0.452 to 0.168)	0.7 (−2.6 to 3.9)
5^g^	−0.235 (−0.442 to −0.029)^c^	−0.7 (−3.8 to 2.4)
6^g^	−0.217 (−0.515 to 0.082)	−0.7 (−5.3 to 3.9)

Abbreviation: BMI, body mass index.

^a^ Estimates are jointly controlled for race and ethnicity, age, gender, insurance status, and city by year fixed effects, as well as intraclass correlation within patients.

^b^ Calculated as weight in kilograms divided by height in meters squared.

^c^ *P* value ≤.05.

^d^ Other race refers to American Indian or Alaskan Native, multiracial, and other races and ethnicities not listed or unknown.

^e^ Refers to primary residence with neighborhood household poverty greater than 20% based on US Census data.

^f^ Year of tax implementation is 2015 in Berkeley and 2017 for Albany, San Francisco, and Oakland.

^g^ Berkeley had 2 additional years of data. Estimates presented are calculated for Berkeley only.

## Discussion

This study examined the association of SSB taxes with BMI and proportion with overweight or obesity in a diverse cohort of 1 044 274 adults living in demographically matched California cities with and without SSB taxes and investigated variation in those associations across specific demographic groups. We hypothesized that cities with SSB taxes would have lower BMI after the tax implementation compared with the control cities. We found that SSB taxes had the most pronounced associations with BMI in specific demographic groups, including adults aged 20 to 39 years, the age group with the highest SSB consumption in adulthood,^19^ and in the city of Berkeley, the first US city to implement an SSB excise tax. SSB taxes were also associated with lower BMI among adults who were female or White. In the entire cohort, however, when considering all taxed cities and all demographic groups together, there was not a statistically significant population-level change in BMI or in the proportion of adults with overweight or obesity. However, the point estimate for overall change in BMI was in the expected direction and equivalent to about a 0.8-lb reduction in weight for a 5′4” woman with a BMI of 27. Our results suggest that the hypothesized benefits of SSB taxes are not equally distributed among residents in cities with SSB taxes.

We hypothesized that SSB taxes are associated with weight through a sequence of increased consumer prices, decreased purchasing, and decreased consumption of SSBs but also by funding nutrition programs and raising awareness about the health harms of SSBs.^20,21,22^ Evidence has shown that since the implementation of SSB taxes, the consumer price of SSBs in Oakland, San Francisco, and Berkeley had, respectively, increased by 0.92 cents per ounce,^4^ 1.00 cent per ounce^4^ and 0.47 cents per ounce.^5^ Moreover, research has also found that the volume of SSB purchases had decreased in Oakland^23^ and Berkeley,^24^ and in Berkeley, reported SSB consumption decreased by 52.5% 3 years after the tax.^25^ Correspondingly, we observed a significant decrease in adult BMI in Berkeley relative to comparison cities. However, despite there also having been decreased SSB purchasing in Oakland and San Francisco, this may not have translated into a population-level reduction in BMI among adults of all ages in these 4 cities collectively. Influences on body weight are multifaceted, and in addition to SSB consumption, there are an abundance of risk factors associated with overweight and obesity, ranging from overall diet quality to the built environment.^26^ Cities with SSB taxes have acknowledged these factors by allocating tax revenue to improve health (eg, access to healthy food) and human and community capital (eg, child development and community infrastructure). Albany, Berkeley, and San Francisco, respectively, invested 100%, 92%, and 79% of SSB tax revenue to health-related goals, while Oakland divided allocations relatively evenly between human and community capital and health.^22^ Berkeley, in particular, created a commission of Berkeley residents to identify community needs and priorities and advanced funds for programs as early as 6 months after SSB tax implementation.^20^ Berkeley’s implementation of SSB taxes may contribute to its observed association with BMI. In other cities, these initiatives may require substantial time to yield results, during which prevailing structural factors may continue to exert significant influence on overall rates of overweight and obesity. However, further economic analysis is needed to understand the impacts of these tax-supported programs on health and weight.

Seattle and Philadelphia have also conducted evaluations of a citywide SSB excise tax and its association with BMI among adults in the US. The most notable difference was tax rate: both cities implemented higher tax rates (1.75 and 1.5 cents per ounce in Seattle and Philadelphia, respectively) than the California cities in this study (1.00 cent per ounce). In Seattle, the SSB excise tax was associated with a small but statistically significant decrease of 0.031 in the annual change in BMI among adults 2 years after the tax. Seattle’s stratified analysis showed beneficial associations among women, adults residing in low-poverty neighborhoods, and those in areas with higher educational attainment. In Philadelphia, there was limited evidence of a decrease in BMI at the end of the 3-year follow-up period, and no differences between demographic subgroups. As in Seattle, our results supported an association between SSB taxes and BMI among women, but we also found an association among young adults and White adults. All 3 studies found no association between SSB taxes and BMI among demographic groups that tend to be higher consumers of SSBs (ie, males, Hispanic and non-Hispanic Black adults, and lower-income adults^19,27,28^). Concerningly, men, Hispanic and non-Hispanic Black adults, and low-income adults not only exhibit the highest prevalence of overweight and obesity,^29^ type 2 diabetes,^30^ and cardiovascular disease,^31^ but also face disproportionate targeting of SSB advertisements.^32^ Moreover, we found a very slight increase in the proportion of adults with overweight or obesity (but not BMI) in neighborhoods where over 20% of households live in poverty. These findings suggest that these SSB taxes may not alone impact obesity across all age groups or address adulthood disparities in BMI, despite their impact on certain demographic groups.

Recent simulation studies projected that SSB taxes could reduce mean BMI among adults from 0.08 with a 1 cent per ounce tax over 2 years^33^ to 0.197 with a 2 cents per ounce tax over 10 years.^34^ While the magnitude of reductions appear small, on a population-level, it is estimated that a mean reduction of 0.197 among adults in California over 10 years will result in 266 000 cases of obesity prevented by 2032 and 114 000 quality-adjusted life years gained.^34^ Thus, despite insignificant findings for the overall cohort, the magnitude of associations of the SSB tax within key demographics could cause population-level BMI changes over time. Our study examined 4 to 6 years of data after the tax. Yet, this follow-up time could be too short to detect a population-level impact on our study outcomes. The decrease in BMI among young adults between 20 to 39 years with the greatest decrease of 0.295 among those aged between 20 and 25 years may indicate the long-term potential of the SSB excise tax. Obesity in young adulthood only or in both young and middle adulthood is associated with a 40% to 90% increased risk of mortality compared with individuals without obesity.^35^ As this age cohort grows older, reduced consumption of SSBs (potentially beyond current evidence of lowered SSB consumption associated with older age) and lower BMI may lead to decreased prevalence of health conditions associated with overweight and obesity in the population over time. Additionally, in a separate study^9^ examining the children and youths of this cohort, SSB taxes were associated with notably lower BMI percentiles, and studies indicate that the dietary patterns and body composition established during childhood are associated with those observed in adulthood.^26^

### Limitations

Our study had limitations. First, the cohort was large and diverse but limited to KP members with at least 1 BMI measurement before and after SSB tax implementation. While this could introduce selection bias, the extent would be minimal with less than 5% of the original study population being excluded due to this criterion. Second, the cohort was limited to patients residing in cities with demographics comparable with those of the 4 cities with SSB taxes. On average, these 4 cities have higher wealth, larger populations, and greater levels of education compared with most other cities in California,^36^ and this resulted in notable differences in race and ethnicity, age, and educational attainment in our matched cities. While we adjusted for these covariates, there may be unmeasured confounding given the notable differences in pretax implementation BMI between cities with and without SSB taxes. Third, cities could have implemented other interventions or policies that affect adult weight over the study period and the magnitude of the association may have been underestimated. Finally, our analysis only examined the total association of SSB taxes on BMI. Data on SSB intake is not available in the EHR, and we did not measure or examine individual-level mechanisms of this relationship (eg, SSB purchasing and consumption) and recommend future studies to examine potential mediators.

### Conclusions

In conclusion, study findings support the association of SSB taxes on BMI in specific demographic groups, including adults aged 20 to 39 years who are the heaviest consumers of SSBs and in Berkeley, the first US city to adopt an SSB tax. However, some of the groups for which we observed significant declines in BMI are not aligned with those at highest risk for overweight or obesity and cardiometabolic conditions. Future research should examine the mechanisms of these associations to inform how SSB taxes could be more equitable for weight-related outcomes.

## Notes

Supplement 1. **eAppendix. **Technical Details of the Statistical Approaches **eTable 1. **List of the 44 Study Cities
eReferences.
**eTable 2. **Characteristics of Exposure and Control Cities, Based on City-Level Data From the American Community Survey (ACS) **eFigure. **Map of Sugar-Sweetened Beverages (SSB) Tax and Control Cities in Kaiser Northern California

Supplement 2.
Data Sharing Statement
