# Supplementary material for: City-Level Sugar-Sweetened Beverage Taxes and Changes in Adult Body Mass Index
Source: JAMA Netw Open. 2025 Jan 24;8(1):e2456170. doi: 10.1001/jamanetworkopen.2024.56170 (PMC11762258; doi:10.1001/jamanetworkopen.2024.56170)
Supplement: Supplement 1. — eAppendix. Technical Details of the Statistical Approaches eTable 1. List of the 44 Study Cities eReferences. eTable 2. Characteristics of Exposure and Control Cities, Based on City-Level Data From the American Community Survey (ACS) eFigure. Map of Sugar-Sweetened Beverages (SSB) Tax and Control Cities in Kaiser Northern California [file jamanetwopen-e2456170-s001.pdf]

Supplemental Online Content

Liu EF, Young DR, Sidell MA, et al. City-level sugar-sweetened beverage taxes and changes in adult body mass index. *JAMA Netw Open*. 2025;8(1):e2456170. doi:10.1001/jamanetworkopen.2024.56170

eAppendix. Technical Details of the Statistical Approaches

eTable 1. List of the 44 Study Cities

eReferences.

eTable 2. Characteristics of Exposure and Control Cities, Based on City-Level Data From the American Community Survey (ACS)

eFigure. Map of Sugar-Sweetened Beverages (SSB) Tax and Control Cities in Kaiser Northern California

This supplemental material has been provided by the authors to give readers additional information about their work.

© 2024 Liu EF et al. *JAMA Network Open*.

eAppendix. Technical Details of the Statistical Approaches

1. The study city matching procedure

Data used for matching

The California sugar sweetened beverage (SSB) Tax Study is a policy evaluation study using a natural experiment design. The intervention arm consisted of four California cities that adopted the SSB excise tax between 2015 and 2017 (Albany, Berkeley, San Francisco, and Oakland). The four intervention cities are all located in the greater San Francisco Bay area with distinct city-level characteristics. Potential control cities included 328 incorporated cities in California, where health care is covered by Kaiser Permanente (KP) and no SSB excise tax was in effect between 2009 and 2020. Seventeen city-level covariates were collected: total population, population density , % population having KP membership, % males, % females, % population in each of the four age strata (≤ 19, 20-44, 45-64, ≥ 65), % population in each of the four race/ethnicity categories (Hispanic of all races, non-Hispanic African American, non-Hispanic white, non-Hispanic Asian and others), % population living below poverty line, and % population in each of the three education attainment levels (high school diploma/GED or lower, some college or associate degree, bachelor degree or higher). Except for % population having KP membership, all other covariates are public information using 5-year averages of U.S. Census American Community Survey prior to SSB tax implementation years.

Matching

Weighted Euclidean distance metrics were calculated using 14 of the 17 covariates by removing a redundant category from three factors (% males, ≥ 65 years old, bachelor's degree or higher) and after standardization (i.e., dividing the raw value by the population standard deviation) using the formula

$$d(p,q) = \sqrt{\sum_{i=1}^{14} w_i (q_i - p_i)^2}$$

where $p=(p_1, p_2, \ldots, p_{14})$, $q=(q_1, q_2, \ldots, q_{14})$ are two points in the 14-dimension Euclidean space, q_i, p_i, $i=1, \ldots, 14$ are standardized matching dimensions, and w_i = weight for dimension i.

Weights were assigned to down weight percentages in the same factor (e.g., the three percentages for age strata each had a weight of .333) so that the total weight of a factor with more than one level was 1. All pairwise distance metrics between a treatment city and a potential control city were calculated. Each intervention city was matched to 10 control cities with generally the shortest distance metrics. Minor ad hoc adjustments were made to avoid overlap in selected control cities among different intervention cities and to ensure no control cities bordered any intervention cities. Specifically, first we excluded any matched city that was geographically adjacent to the intervention city and replaced it with the next available control city from the list ordered by Euclidean distance. Next, we examined controls to ensure that each control city was only matched to one intervention city. If a city was matched to multiple intervention cities, the match with the smallest corresponding Euclidean distance was maintained while the other was replaced with that intervention city's next available control city from the list ordered by Euclidean distance. The final list of intervention and matched control cities is listed in **eTable 1.**

Balance assessment

Balance assessment was ascertained by examining the absolute standardized difference between the mean value (SMD) for matched control cities and the value of each covariate in the intervention city. Due to the small sample size and the finite study population of California cities, some covariates have moderately large SMD. We conducted a Monte Carlo simulation to assess the relative goodness of balance. Specifically, 100,000 ideal random samples of California cities were drawn (4 treated cities and 40 control cities in each random sample). The SMD of our chosen study sample is smaller than or close to

© 2024 Liu EF et al. *JAMA Network Open*.

the median SMD of the 100,000 ideal random samples. Details of the simulations are reported elsewhere. (Han and Sidell, 2024) Therefore, we concluded that in relation to an ideal simple randomization, the chosen study sample had acceptable levels of covariate balance.

eTable 1. List of the 44 Study Cities

Tax City	Matched Comparison Cities
Albany	San Ramon, Dublin, Pleasanton, Santa Clara, San Rafael, Burlingame, Walnut Creek, Belmont, Claremont, Lafayette
Berkeley	Davis, Pasadena, Loma Linda, Sunnyvale, Fullerton, San Mateo, Tustin, Signal Hill, South Pasadena, Placentia
Oakland	Sacramento, Moreno Valley, Pittsburg, Stockton, Elk Grove, Rialto, Hayward, Fontana, San Leandro, Corona
San Francisco	San Jose, San Diego, Irvine, Anaheim, Fremont, Riverside, Long Beach, Chula Vista, Glendale, Bakersfield

2. The stratified difference-in-differences (DID) method

Overview

The difference-in-differences (DID) method is a widely applied causal inference method, conventionally operationalized through an Analysis of Covariance (ANCOVA) longitudinal regression model. The key assumption of the DID method was that in the absence of any treatment, the trajectories of the mean outcome would have been parallel between any study arms (Lechner, 2011). We adopted the DID method as the main analytic approach in this paper. However, the massive study sample from KP's electronic health record (EHR) imposed a great challenge. Standard ANCOVA longitudinal regression models, including the many variants, could not pass the general falsification and validation tests for model specification and the fundamental parallel trajectory assumption. The standard ANCOVA regression might yield biased estimates for the SSB tax treatment effects as well as potentially false significance. To ensure unbiased estimates and avoid type I errors, we first stratified the full sample into independent subsamples of blocks. In each block, observed covariates were completely homogenous or almost so. The sample size in each block was reasonably small. Thus, adjusting for covariates was either not needed or could be adequately done within each block. Second, the trajectories of the mean outcome were fully nonlinear in each block. These two adjustments made the analysis in each block adequately verified. Finally, results from all blocks were aggregated to form the overall estimates and subgroup estimates. Details of these steps were as follows.

Data, conventional DID, and validation and falsification tests

We first introduced the standard notation for unsynchronized longitudinal data: the outcome data were denoted as $Y(i,t_{i,j})$ for patient i=1,…,N, $N \approx 3.9\times10^5$ and in year $t_{i,j}$. Patient i had repeated measurements in a sequence of years $\{t_{ij}: t_{i,1}, \ldots, t_{i,Ti}\}$. All years $t_{i,j}$ ranged between 1 and 10, where the first six years were pre-tax and the last four years were post-tax (ignoring the different timeline for Berkely for the sake of brevity). We required that the number of measurements in patient i be $Ti \geq 2$, the first measurement time $t_{i,1}<7$, and the last measurement time $t_{i,Ti} \geq 7$. Except for these requirements, all patients could have different numbers of measurement and different measurement times. The total number of measurements was $\sum_{i=1}^{N} Ti \approx 2.2\times10^6$. Let Z_i = 1 or 0 denote whether a patient in a treated city, and X_i denote the observed baseline covariate vector (birth year, sex, race/ethnicity, insurance status, and specific city of residence). Let I{condition} denote a dummy variable for the condition within the brackets. With this notation system, a basic ANCOVA model had the following mean function

$$E[Y(i,t_{i,j})] = \alpha_1 + Z_i\alpha_2 + \gamma t + \mu t Z_i I\{t>6\} + X_i\beta, \quad (1)$$

© 2024 Liu EF et al. *JAMA Network Open*.

where the parameter of interest was μ (i.e., the coefficient for the time by condition interaction). Other parameters in the mean function include the intercept α_1 for mathematical purposes, the baseline difference between arms $Z_i\alpha_2$, the adjusted covariate effect $X_i\beta$, and the common trajectory γt, which would be followed by everyone in the absence of treatment. The variance components of the ANCOVA model besides Equation (1) included the random measurement error, the serial correlation within a patient, and potentially the clustering within a city, where the last two error components could be modeled by random effects, covariance terms, or the fixed-effect approach. Each approach further had many technical variants. In the sequel, we focused on the mean function and omitted most tedious technical details in variance components.

The main challenge for the DID approach was encountered in the two types of validation and falsification tests. First, a goodness-of-fit test checked if the working model underfitted the data. Due to the massive sample size and highly heterogeneous patients (390k patients, 2.2 million records), it was almost certain that a conventional parametric regression could not adequately fit the data. Minor enrichments of model (1), such as short-term shock, two-way interactions, parabolic or cubic time trends, or regression spline in time trends, were of little to no help. For example, a slightly enriched model for (1) was to replace the parameter of interest $\mu t Z_i I\{t>6\}$ by two terms $\mu_1 Z_i I\{t>6\} + \mu_2 t Z_i I\{t>6\}$, i.e., an instant shock and a long-term linear effect. Not surprisingly, the enriched model had significantly better goodness of fit than the working model by any reasonable goodness-of-fit test or model selection/comparison criteria. However, the enriched model itself also substantially underfitted the data and could use further expansion.

Sometimes a model's goodness-of-fit was deemed as a technical concern rather than a substantive jeopardy. However, the placebo test for DID was always deemed as crucial to partially justify its validity. The rationale of the placebo test was to conduct the DID analysis in the pre-tax period only by setting a fake and shortened post-tax period, e.g., using years 1 to 5 as pre-tax and year 6 as the fake post-tax period. Since no treatment was in effect, the placebo test was expected to be insignificant if the parallel trajectory assumption was not violated. Nevertheless, it was almost certain that any conventional parametric regression to DID could not pass the placebo test. Lastly, the massive dataset also made it challenging to fit most conventional models and perform these tests computationally: a single mixed-effect model could take more than several days to fit in SAS Studio Enterprise 9.4.

Our stratified and saturated DID

Stratification: as recommended by the official user manual of SAS/STAT software, we partitioned the sample into mutually exclusive subsamples or blocks to reduce the total computational burden, where blocks were defined by intervention city, race and ethnicity, sex, birth years, and insurance status jointly. For example, a block was Hispanics, males, having Medicaid or other public health insurance, born between 2005 and 2006, living in San Francisco or its 10 matched control cities. Another block was white, females, any insurance, born between 2006 and 2010, living in Oakland or its 10 matched control cities. Each block had a reasonably small sample size, usually between 10,000 to 100,000 records. Moreover, the observed covariates were either constant or had minor differences within each block so that goodness-of-fit for covariates was no longer a concern.

Saturated DID: within each block there was still the daunting task to adequately model the mean trajectories to pass the placebo test. We employed a fully nonlinear and saturated parametrization based on the robust DID approach in the econometric literature (Conley and Taber, 2011; Rambachan and Roth, 2023). Let $Y^{(g)}_{(i,k,j)}$ denote the outcome for patient i in city k at time j, $1 \le j \le 10$, k=1 for the treated city and k=2,…11 for control cities, and the superscript (g) denoted a distinct block independent of all other blocks. Note that all blocks had exactly 11 cities as presented here. The saturated DID for block g is

$$E[Y^{(g)}_{(i,k,j)}] = \lambda^{(g)}_{k,j} + X_i^{*(g)}\beta^{(g)}. \qquad (2)$$

where each city had a fully flexible trajectory $\lambda^{(g)}_{k,1}, \lambda^{(g)}_{k,2}, \ldots, \lambda^{(g)}_{k,10}$, and the term $X_i^{*(g)}\beta^{(g)}$ adjusted for the few remaining non-constant covariates. Statistically, the 110 distinct parameters $\lambda^{(g)}_{k,j}$ in block g were

© 2024 Liu EF et al. *JAMA Network Open*.

the highest-order interaction term that could be applied to the mean function. All variance components were modeled separately for each block as well. Thus, the collection of all block-level models composed a very large-scale parametrization with roughly 110 x 104 ≈ 1.1 $\times 10^4$ parameters, resulting in a "n-p ratio" of roughly 190, i.e., 190 data points per unknown parameter for estimation. (Note: the actual number of parameters is slightly more than this due to the variance components and the few covariate terms). We used the following placebo test for model (2)

$$H_0: \left[\lambda_{1,6}^{(g)} - \tfrac{1}{5}\sum_{j=1}^{5}\lambda_{1,j}^{(g)}\right] - \left[\sum_{k=2}^{11} v_k^{(g)}\lambda_{k,6}^{(g)} - \tfrac{1}{5}\sum_{j=1}^{5}\sum_{k=2}^{11} v_k^{(g)}\lambda_{k,j}^{(g)}\right] = 0,$$

where control cities had weights $v_k^{(g)}$, k=2, …,11. These weights were equal to 0.1 by default (i.e., 10 controls equally weighted). Causal effect contrasts were estimated only if the placebo test could pass, i.e. fail to reject H_0. In the event that the placebo test failed to pass, we made one or more of the following adjustments, including splitting the block, combining the block with an adjacent block, or combining and re-splitting the block with adjacent block(s). Failure to pass the placebo test might also result from one or more control cities whose trajectories were substantially different from other control cities and the treated city. In these cases, we set $v_k^{(g)} = 0$ to exclude these control cities and adjusted the weights or the remaining control cities in this block. For example, if we decided to exclude one control city in a block, then this excluded city's weight was 0 and the other 9 control cities weight was changed to 0.1111.

After passing the placebo test, the causal effect for a post-tax year t, $10 \geq t \geq 7$, was the following linear contrast

$$L_t^{(g)} = \left[\lambda_{1,t}^{(g)} - \tfrac{1}{6}\sum_{j=1}^{6}\lambda_{1,j}^{(g)}\right] - \left[\sum_{k=2}^{11} v_k^{(g)}\lambda_{k,t}^{(g)} - \tfrac{1}{6}\sum_{j=1}^{6}\sum_{k=2}^{11} v_k^{(g)}\lambda_{k,j}^{(g)}\right].$$

The causal effect for the overall effect across four post-tax years was the following linear contrast

$$L^{(g)} = \tfrac{1}{4}\sum_{t=7}^{10} L_t^{(g)}.$$

All causal effect contrasts and the placebo test statistics were estimable under the general linear hypothesis inference framework (McLean et al., 1991).

Aggregation operation

Aggregated point estimates and standard errors were calculated by taking the overall mean of the block estimates weighted by proportion of distinct subjects in the intervention city $w^{(g)}$,

$$L_t = \sum_g w^{(g)} L_t^{(g)}, \text{ and } L = \sum_g w^{(g)} L^{(g)}.$$

Subgroup (race/ethnicity, age, and sex) point estimates were calculated using the same method above but with the summation over blocks sharing the common subgroup characteristics and weights standardized to sum to one among these blocks. By statistical independence among blocks, the SE of the contrasts L_t, L, and subgroup effects were square roots of the sum of squared SEs from all blocks involved. We applied the Wald's z test inference to calculate 95% confidence intervals and p-values for the aggregated results.

The full technical details and software codes of the stratified saturated DID approach will be reported elsewhere and are available upon requests. All data analysis by the DID approach was conducted using PROC MIXED in SAS Studio Enterprise version 9.4 (SAS Institute Inc., Cary, NC, USA).

eReferences

B. Han and M. A. Sidell (2024). Pseudo p-values for assessing covariate balance in a finite study population with application to the California sugar sweetened beverage tax study. arXiv:2404.09960

Lechner M. (2011). The estimation of causal effects by difference-in-difference methods. *Foundations and Trends in Econometrics*. 4(3):165-224.

© 2024 Liu EF et al. *JAMA Network Open*.

McLean, R. A., Sanders, W. L., and Stroup, W. W. (1991). A unified approach to mixed linear models. *American Statistician* 45:54–64.

Conley, T.G. and Taber, C.R., 2011. Inference with "difference in differences" with a small number of policy changes. *The Review of Economics and Statistics*, 93(1), pp.113-125.

Rambachan, A. and Roth, J., 2023. A more credible approach to parallel trends. *Review of Economic Studies*, 90(5), pp.2555-2591.

© 2024 Liu EF et al. *JAMA Network Open*.

eTable 2. Characteristics of Exposure and Control Cities, Based on City-Level Data From the American Community Survey (ACS) [a]

Characteristic	Albany		Berkeley		Oakland		San Francisco		Total	
	Exposure (N=1) [b]	Controls (N=10) [c]	Exposure (N=1) [b]	Controls (N=10) [c]	Exposure (N=1) [b]	Controls (N=10) [c]	Exposure (N=1) [b]	Controls (N=10) [c]	Exposure (N=4) [c]	Controls (N=10) [c]
**Population**										
Total (10k)	1.91	6.12 (2.80)	1.13	7.26 (5.39)	40.49	19.03 (12.11)	8.46	47.96 (38.99)	34.59 (37.17)	20.09 (26.17)
Density (1000 persons/mile)	3.50	3.94 (1.35)	6.41	5.84 (1.25)	5.20	4.18 (0.89)	3.65	4.92 (2.10)	4.69 (1.38)	4.72 (1.60)
**KP Membership [d] (%)**	0.36	0.32 (0.06)	0.26	0.19 (0.05)	0.34	0.34 (0.09)	0.26	0.21 (0.08)	0.30 (0.05)	0.26 (0.09)
**Female (%)**	0.51	0.51 (0.01)	0.51	0.51 (0.01)	0.51	0.51 (0.01)	0.49	0.51 (0.01)	0.51 (0.01)	0.51 (0.01)
**Age (%)**										
19 or less	0.29	0.25 (0.04)	0.21	0.23 (0.05)	0.23	0.30 (0.04)	0.15	0.27 (0.04)	0.22 (0.06)	0.26 (0.05)
20-44	0.38	0.34 (0.05)	0.44	0.42 (0.06)	0.40	0.36 (0.02)	0.45	0.38 (0.02)	0.42 (0.03)	0.37 (0.05)
45-64	0.24	0.27 (0.03)	0.22	0.24 (0.03)	0.25	0.24 (0.03)	0.26	0.24 (0.02)	0.24 (0.02)	0.25 (0.03)
65+	0.10	0.14 (0.06)	0.12	0.11 (0.03)	0.12	0.10 (0.02)	0.14	0.11 (0.02)	0.12 (0.02)	0.12 (0.04)
**Race/Ethnicity (%)**										
Asian	0.27	0.25 (0.11)	0.20	0.25 (0.08)	0.16	0.17 (0.10)	0.34	0.22 (0.16)	0.24 (0.08)	0.22 (0.12)
Black	0.05	0.03 (0.02)	0.09	0.06 (0.06)	0.26	0.12 (0.04)	0.05	0.05 (0.03)	0.11 (0.10)	0.07 (0.05)
Hispanic	0.12	0.15 (0.07)	0.11	0.25 (0.10)	0.26	0.43 (0.17)	0.15	0.36 (0.18)	0.16 (0.07)	0.30 (0.17)
White	0.49	0.52 (0.11)	0.56	0.39 (0.08)	0.27	0.24 (0.09)	0.41	0.34 (0.13)	0.43 (0.12)	0.37 (0.14)
**Household income below poverty (%)**	0.11	0.07 (0.03)	0.19	0.13 (0.06)	0.20	0.16 (0.05)	0.13	0.15 (0.04)	0.16 (0.05)	0.13 (0.06)
**Education (%)**										
High School or Less	0.10	0.19 (0.05)	0.13	0.24 (0.09)	0.36	0.46 (0.09)	0.25	0.35 (0.11)	0.21 (0.12)	0.31 (0.14)
Some College	0.17	0.25 (0.02)	0.18	0.25 (0.04)	0.25	0.32 (0.03)	0.20	0.28 (0.05)	0.20 (0.04)	0.27 (0.05)
College Degree or Higher	0.73	0.56 (0.06)	0.70	0.51 (0.13)	0.39	0.22 (0.08)	0.54	0.37 (0.15)	0.59 (0.16)	0.42 (0.17)

[a] Average of 4 years of ACS 5-year averages starting with 4 years prior to exposure year (example exposure year is 2017, value is average of ACS 5-year averages for 4 years starting with 2013.
[b] Data are mean values for exposure cities.
[c] Data are mean (SD).
[d] Percent of the total population that are members of Kaiser Permanente in the first quarter of the year.

© 2024 Liu EF et al. *JAMA Network Open*.

eFigure. Map of Sugar-Sweetened Beverages (SSB) Tax and Control Cities in Kaiser Northern California

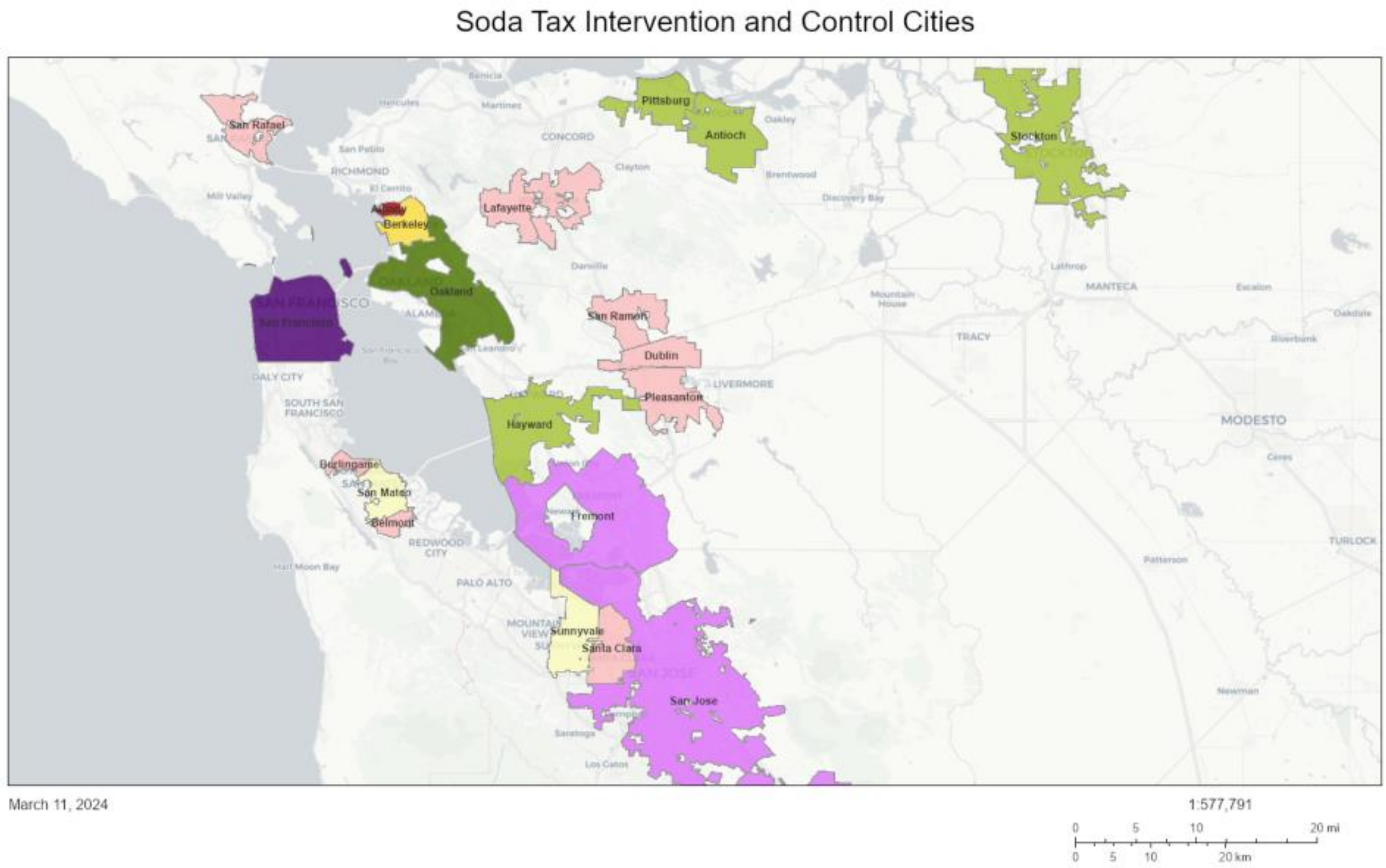

There were 21 control cities were from Kaiser Permanente Southern California (KPSC) with little to no possibility for exposure contamination. The remaining 19 control cities in Kaiser Permanente Northern California (KPNC) were geographically dispersed with no control cities bordering any exposed cities. Above is a regional map of KPNC. The exposed cities are cities along the bay: Albany (red), Berkeley (yellow), Oakland (green), San Francisco (purple). The corresponding control cities in Northern California are shown using a lighter version of the matched tax city's color. Not shown on the map are two Northern California control cities, Sacramento and Davis, which are geographically distant, and the 21 control cities from the KPSC region.

© 2024 Liu EF et al. *JAMA Network Open*.
