# Supplementary material for: City-Level Sugar-Sweetened Beverage Taxes and Changes in Adult Body Mass Index
Source: JAMA Netw Open. 2025 Jan 24;8(1):e2456170. doi: 10.1001/jamanetworkopen.2024.56170 (PMC11762258; doi:10.1001/jamanetworkopen.2024.56170)
Supplement: Supplement 2. — Data Sharing Statement [file jamanetwopen-e2456170-s002.pdf]

Data Sharing Statement

Liu. City-Level Sugar-Sweetened Beverage Taxes and Changes in Adult Body Mass Index. *JAMA Netw Open*. Published January 24, 2025. doi:10.1001/jamanetworkopen.2024.56170

Data
Data available: No

Additional Information
Explanation for why data not available: Data was obtained from electronic health records and contains protected health information. It will not be shared.
